# Reverse migration and the health emergency among Afghan returnees: a call for regional action

**DOI:** 10.1186/s12939-025-02723-9

**Published:** 2025-12-05

**Authors:** Seyed Aria Nejadghaderi, Samira Emadi, Ghulam Raza Mohammadyan, Hamid Sharifi, Majid Fasihi Harandi, AliAkbar Haghdoost

**Affiliations:** 1 HIV/STI Surveillance Research Center, and WHO Collaborating Center for HIV Surveillance, Institute for Futures Studies in Health, Kerman University of Medical Sciences, Kerman, Iran; 2 Knowledge Hub for Migrant and Refugee Health, Institute for Futures Studies in Health, Kerman University of Medical Sciences, Kerman, Iran; 3 Health Services Management Research Center, Institute for Futures Studies in Health, Kerman University of Medical Sciences, Kerman, Iran; 4 Department of Biostatistics and Epidemiology, Faculty of Public Health, Kerman University of Medical Sciences, Kerman, Iran; 5 Institute for Global Health Sciences, University of California, San Francisco, CA USA; 6 Research Center for Hydatid Disease in Iran, Kerman University of Medical Sciences, Kerman, Iran; 7 Social Determinants of Health Research Center, Institute for Futures Studies in Health, Kerman University of Medical Sciences, Kerman, Iran

**Keywords:** Transients and migrants, Afghanistan, Iran, Delivery of health care, United Nations, Public health, Return migration, Reverse migration, Deportation, Migration health, Afghan returnees

## Abstract

The current geopolitical situation in the Middle East suggests a growing trend of reverse migration, where migrants and refugees may be gradually returned to their home countries. This large-scale return, exemplified by the movement of Afghan migrants from Iran and Pakistan and the potential return of Syrians, places immense pressure on the healthcare systems of their native countries, which already face numerous challenges. This manuscript synthesizes literature through 30 June 2025 and primary agency situational and flow reports, including IOM border and flash updates, WHO-EMRO returnee health situation reports, and UNHCR emergency updates, to document the 2025 return surge and its health impacts. The recent mass return of over thousands Afghan migrants from Iran in 2025 can lead to a crisis. Driven by the return process and arduous journeys, these returnees face a profound public health crisis. Physical ailments like malnutrition, dehydration, and infections are prevalent, compounded by psychological trauma including anxiety, depression, and post-traumatic stress disorder. This influx overwhelms Afghanistan’s already fragile and underfunded healthcare system, which struggles with limited resources and infrastructure. Therefore, during the reverse migration and return of migrants, attention to the health systems in the countries of origin is as crucial as it was during the initial migration. Urgent, coordinated international medical aid, psychosocial support, and disease prevention strategies are needed to address this crisis.

## Clinical trial registration

Not applicable.

The contemporary geopolitical landscape is frequently marked by large-scale reverse migration, where populations are returning to their countries of origin. Reverse migration is not only a demographic event, it constitutes a transboundary public-health shock that must be read through conceptual lenses that make visible the social drivers and system vulnerabilities that produce concentrated health harms. This trend is observable in the return of Afghan migrants from Pakistan and Iran, and similar dynamics have been documented elsewhere globally (e.g., pandemic-related returns, post-conflict repatriations), and it is probable that a significant number of Syrians will also return to their home country if greater stability is achieved [1]. Such enforcement actions, as exogenous policy triggers, align with broader global patterns where state decisions exacerbate humanitarian vulnerabilities, as seen in similar large-scale reverse-migration events, such as COVID-19-related returns of informal workers to rural Bangladesh (2020–2023), which strained local health and nutrition services and revealed gaps in rural health-system capacity, the mass displacement and returns associated with the 2021 Afghan crisis, and the protracted Rohingya displacement with failed returns that overwhelmed host health systems in Bangladesh, illustrate that the health effects documented here echo global patterns [2, 3].

For the purposes of this study, we define “reverse migration” as a rapid, large-scale return of migrants to their country of origin within weeks to months, where returns are predominantly involuntary or coerced and produce a concentrated demographic shock to origin-country systems. We define “health emergency” as the presence of acute population health indicators (e.g., malnutrition, dehydration, acute infectious disease cases), demonstrable health-system stress (e.g., facility overcrowding, drug stock-outs, critical humanitarian funding shortfalls), and epidemic-risk conditions (crowding, low immunization coverage). Numbering in the hundreds of thousands, these individuals arrive in states of profound bewilderment and physical exhaustion, underscoring the acute human element of this crisis [1]. This situation creates pressure on the health systems of these nations, which are already grappling with their own significant problems. Beyond the provision of basic necessities, the physical and mental well-being of these returnees is compromised, an aspect frequently overlooked in broader discourse. Moreover, some suggest that states should bear accountability for migrants’ healthcare needs not only during emigration but also upon return [4, 5]. For this reason, just as the health of migrants requires special attention during their emigration, it is necessary to focus on health issues during the process of their return.

Deportation, understood as the involuntary physical removal of individuals from one state’s territory to another’s, has increasingly garnered academic and policy attention [6]. This practice, acknowledged for its brutality, expense, and frequent ineffectiveness in migration management, generates profound post-removal challenges [7]. From a structural vulnerability perspective, deportation functions as a form of structural violence. It is a policy instrument that concentrates social and economic insecurity and thereby produces predictable health consequences (e.g., interrupted chronic care, elevated mental-health morbidity, increased susceptibility to infectious disease) [8]. This structural violence links policy enforcement to clustered harms, increasing vulnerabilities through disrupted social networks and economic precarity. Post-deportation outcomes often render adjustment, integration, or reintegration exceedingly difficult, particularly due to factors such as debts incurred during migration, severed transnational and local ties, the pervasive shame of perceived failure, and community perceptions of “contamination” [6]. Syndemic thinking further suggests that the clustering of malnutrition, infectious disease exposure, and trauma/mental-health conditions in returnee populations will interact to increase morbidity beyond what each condition would cause in isolation [9]. Such multifaceted difficulties impinge upon the health status of returnees. Therefore, this article aims to dissect the causes underlying this extensive population movement, to delineate the specific and alarming public health crisis it has engendered, and to issue a call for robust, targeted medical aid and international support for the Afghan returnees. Similar harms have been observed in forced-return contexts in sub-Saharan Africa where returns exacerbated infectious-disease outbreaks and disrupted chronic-care continuity, and in Latin America where Venezuelan and Haitian returnee surges placed extreme pressure on bordering health systems [10, 11].

We conducted a targeted secondary-data review and synthesis of primary agency situation reports (International Organization for Migration [IOM], WHO, United Nations High Commissioner for Refugees [UNHCR]), corroborated media reporting, and recent peer-reviewed studies addressing deportation, migration and health. Agency reports and situational updates were included if they were published between 1 January and 30 June 2025 and provided primary counts or field assessments relating to returnee numbers or health status. We triangulated quantitative data with qualitative assessments and peer-reviewed analyses, applying inclusion criteria for relevance and excluding non-primary sources. Key agency sources include IOM border monitoring and flash updates, WHO-EMRO returnee health situation reports, and UNHCR emergency updates. Also, we operationalized “reverse migration” and “health emergency” as described above, and mapped empirical observations to a conceptual model that links policies in host countries, rapid forced returns, disrupted continuity of care and acute exposure to food insecurity, clustered physical and mental-health outcomes, and system stress. Where available, we extracted quantitative indicators (e.g., numbers of returnees) and used qualitative agency findings to interpret mechanisms (e.g., reports of family separation, transport conditions). This methodological approach improves conceptual coherence by integrating structural vulnerability and syndemic theory into data synthesis and interpretation.

The magnitude and velocity of Afghan returns from Iran in 2025 represent an unprecedented demographic shift, straining Afghanistan’s already precarious humanitarian infrastructure [12]. Between January 1 and June 29, 2025, the IOM documented 714,572 Afghan migrants returning from Iran, with a staggering acceleration observed in June alone, accounting for over 256,000 arrivals [12]. These figures illustrate what Castles describes as ‘migration shocks,’ where rapid inflows overwhelm state absorptive capacity and reveal underlying structural vulnerabilities [13]. This record-setting influx, reaching over 28,000 individuals crossing in a single day on June 25th and an average daily return of about 20,000 Afghan migrants, overwhelms existing border resources and reception capacities [12]. Overall, more than 1.5 million Afghan refugees or migrants have been sent back to Afghanistan from Iran over seven months of 2025 [14]. The scale of return flows aligns with the literature on forced immobility and crisis mobility, in which state policy decisions act as exogenous triggers of humanitarian system failure [15]. A main characteristic of this migratory wave is its involuntary and undocumented nature; 99% of these returnees lacked formal documentation, and a significant proportion of returnees reported that they were forcibly returned [12]. Furthermore, a demographic shift has been noted, with an increase in the deportation of vulnerable families, including women and children, a departure from earlier trends dominated by single young men. Upon arrival at border crossings such as Islam Qala and Milak, these individuals are often present in states of acute disorientation, exhaustion, and hunger, indicative of severe humanitarian needs [12].

The current health vulnerability among Afghan returnees is attributable to a confluence of policy shifts and pre-existing pressures. Policy announcements and enforcement measures in certain host countries in 2025 contributed to an accelerated pace of returns. These measures occurred alongside longer-standing barriers that left many migrants legally and economically precarious. A primary reason for the accelerated returns is the Iranian government’s directive, issued on March 20, 2025, mandating the departure of all undocumented Afghan migrants by July 6, 2025 [12]. Similar policy-driven return dynamics have occurred in other regions and that the consequent health risks follow common pathways internationally. This policy, viewed within the context of regional geopolitical dynamics, has translated into rapid returns, precluding any opportunity for adequate preparation or health screenings prior to deportation [12]. Despite providing free basic services, such as vaccinations, for Afghan refugees, many continue to underutilize healthcare due to lack of proper documents and fear of deportation, compounding their vulnerability even before being forced out [16]. The journey back to Afghanistan itself has a severe impact on health. Conditions characterized by a lack of food, potable water, and adequate sanitation, as well as prolonged exposure to environmental elements, compromise physiological well-being [12]. Such arduous transit exacerbates pre-existing health conditions and contributes to acute dehydration, fatigue, and susceptibility to various illnesses upon arrival [12]. Beyond direct enforcement, interacting factors, including economic strains, discrimination, regional security pressures, and parallel policies in neighboring states, drive returns and increase health risks through structural vulnerability, where legal precarity concentrates exposure to harms. These drivers echo global patterns, informing cross-regional policy lessons [1, 12].

The mass return of Afghan migrants from Iran has generated a public health crisis, characterized by widespread physical and psychological distress among the returnee population. Physically, individuals present with acute malnutrition, severe dehydration, and diarrheal diseases, largely attributable to inadequate sanitation and unsafe water sources encountered during their displacement and upon arrival [12]. Respiratory infections, including colds, influenza, and pneumonia, are also prevalent, often resulting from prolonged exposure to environmental conditions during transit [12]. Furthermore, pre-existing chronic conditions, such as diabetes and heart disease, are exacerbated due to the abrupt cessation of medication and lack of continuous medical care. Children are particularly at risk as Afghanistan’s routine immunization coverage remains low, and many returnee children arrive without up‑to‑date vaccinations, increasing their vulnerability to different vaccine‑preventable diseases. Beyond physical ailments, the psychological impact of forced displacement, the trauma of deportation, and feelings of fear, loss, and uncertainty contribute to a significant mental health burden [17]. It was reported that deported migrants experienced significantly more mental health issues compared to other returnee groups [17]. Moreover, the trauma witnessed or experienced by children during forced displacement can lead to long-term developmental and psychological distress. Syndemic theory shows how these co-occurring physical and mental conditions interact synergistically, worsening outcomes beyond additive effects and straining systems already weakened by structural vulnerabilities [9].

In terms of theoretical frameworks, structural vulnerability explains how immigration enforcement, legal precarity, poverty, and social stigma concentrate risk among undocumented Afghans in host countries and upon return [8]. Syndemic logic explains why co-occurring conditions (infectious disease, malnutrition, trauma) interact to produce worse outcomes collectively than each would alone [9]. Health-systems resilience/political-economy analysis explains why Afghanistan’s existing system, shaped by decades of conflict, governance changes, and underfunding, is poorly positioned to solve the shock of mass returns [18]. Together, these explains how policy actions in host countries produce predictable biosocial harms in origin countries.

This humanitarian influx also has public health risks to the wider community. The concentration of vulnerable individuals in crowded, often unsanitary reception centers or makeshift settlements creates fertile ground for the transmission of communicable diseases, including cholera and tuberculosis. This presents a threat not only to the returnees themselves but also to the already strained host communities. Certain demographic groups face heightened health risks. Women, for instance, are particularly susceptible to complications related to maternal health and are disproportionately affected by gender-based violence. Children face elevated risks of severe malnutrition, developmental trauma, and lack of essential vaccinations [12]. Similarly, the elderly and individuals with disabilities encounter exacerbated health challenges due to their specific needs and limited access to support services [12].

Afghanistan’s healthcare infrastructure, already fragile from decades of conflict and economic instability, is now on the verge of collapse under the pressure of the surging returnee population. The current humanitarian response to the returnee crisis faces critical funding gaps; the 2025 Humanitarian Needs and Response Plan, requiring $2.42 billion, has secured only 22.2% of its target [1]. This profound underfunding directly translates into a severe strain on limited health facilities, personnel, and medical supplies, pushing them beyond their operational capacity and impacting both returnees and the existing local populations [12]. Moreover, pre-existing disparities in healthcare access persist across Afghanistan’s regions, with financial constraints and equipment shortages impairing the functionality of healthcare facilities, particularly in the Northeastern and Western provinces [19]. Furthermore, the long-standing shortage of female healthcare providers and restrictions on women’s movement can continue to limit access to essential services [19]. These system strains shows structural vulnerability at the macro level, where chronic underfunding and gender barriers causally interact with return shocks to amplify syndemic risks [9].

Afghanistan’s health system remains fragile that have disproportionately affected the availability and use of services for women and children. National health-facility mapping and service-availability monitoring indicate major gaps in essential service coverage and persistent geographic inequities in service readiness. Systemic contractions disproportionately reduce access to maternal, newborn and child health services and routine immunization [20]. There are shortages of female health workers, supply chain disruptions for essential maternal medicines and supplies, and policy-driven barriers that limit women’s mobility and access to care; together these factors have led to measurable declines in service utilization and worsening maternal and neonatal outcomes in many settings [21]. Policy-driven return flows into a health system weakened by funding cuts, workforce shortages, and gender-specific access barriers are likely to produce both immediate clinical burdens and medium-term declines in maternal and child health and mental-health outcomes unless targeted interventions are deployed rapidly. Consequently, interventions, such as antenatal/postnatal care, skilled birth attendance, catch-up immunization for children, nutrition screening and treatment, provision of female-led services where possible, and integrated mental-health and psychosocial support tailored to women and children in reception and community settings, are necessary [21].

Addressing the health crisis among Afghan returnees necessitates an immediate and comprehensive response. There is a requirement for the provision of immediate life-saving assistance at border points and within transit facilities [12]. This includes the rapid deployment of basic medical care, essential pharmaceuticals, and rapid diagnostic kits to identify and treat prevalent illnesses. Concurrently, emergency food and nutrition support are important to treat acute malnutrition and dehydration observed upon arrival [12]. Furthermore, psychosocial support, trauma counseling, and mental health services must be integrated into primary healthcare provisions [12, 17]. To mitigate the risk of epidemics, the establishment of disease surveillance systems and the implementation of vaccination campaigns are important. Concomitantly, water, sanitation, and hygiene interventions are essential to improve living conditions and prevent the outbreak and spread of communicable diseases in crowded reception and settlement areas [19]. Responses should be coordinated regionally, respect local sovereignty, and avoid politicized language that could be used to instrumentalize humanitarian needs. To operationalize these interventions, we recommend a three‑phase policy roadmap: Phase 1: rapid needs assessment at Islam Qala and Milak; Phase 2: regional referral and care pathways linking border clinics to provincial hospitals particularly in Herat; Phase 3: real‑time health monitoring via a centralized WHO/IOM digital dashboard. International donors must meet the funding targets for Afghanistan’s humanitarian response, explicitly prioritizing health and medical services to bridge the current critical funding gap [1]. These interventions are designed both to reduce immediate biosocial interactions that drive syndemic amplification and to strengthen short-term absorptive capacity within a resilience framework. A collaboration among UN agencies, non-governmental organizations, and local authorities is necessary to ensure the efficient and equitable delivery of health services across all affected populations [12]. While the immediate focus remains on health, efforts should also encompass the broader need for returns and support for long-term reintegration, which includes sustained access to ongoing health services within their new communities [12].

We recommend a response that prioritizes life-saving care at border crossings and rapidly transitions to reintegration supports: immediately deploy standardized triage and mobile emergency medical teams, essential medicines and rapid diagnostics, screening with outpatient/inpatient malnutrition services, and trauma-informed psychosocial first aid at reception sites. Concurrently, the followings should be considered: safe water, gender-segregated latrines, hygiene kits and implement targeted catch-up vaccination campaigns for children and routine immunization assessments; within 2–12 weeks establish regional referral pathways linking border clinics to provincial hospitals, ensure continuity packages for maternal and child health, as well as chronic disease management, and roll out cash-based assistance and education continuity measures to reduce negative coping; over the medium term (3–12 months) invest in health-system resilience, formalize legal/documentation, and multi-year donor funding. All interventions should be implemented with sensitivity to regional political dynamics, coordinated through neutral humanitarian leadership, and informed by comparative global experiences.

The study has some limitations. We relied on secondary agency reports and rapidly produced situational updates. While these are the best available primary field data in an emergency, they may undercount certain flows or underreport health conditions that require facility-level surveillance. The urgency of the event precluded primary clinical surveillance by the authors. We also acknowledge that migration motives are often complex. While agency reports indicate a very large proportion of returns were forced or effectively coerced following policy deadlines in host countries, other factors (e.g., economic, seasonal, family-related) can contribute. Nevertheless, the temporal clustering and contemporaneous policy enforcement make the recent surge best explained principally by host-country enforcement actions. We therefore present policy recommendations as pragmatic, evidence-informed steps while calling for more systematic field surveillance and longitudinal study of returnee health outcomes.

In conclusion, the mass return of Afghan migrants from Iran is part of a broader regional trend of reverse migration that has precipitated a health crisis that can threaten an already fragile nation. The severe health consequences, encompassing both physical ailments and psychological trauma, coupled with Afghanistan’s underfunded healthcare capacity, demand action. To address this, UNHCR should lead registration and protection efforts, establishing reception centers, ensuring legal documentation, and coordinating shelter provision; WHO should spearhead public health assessments, launch targeted vaccination campaigns, and support the scaling‑up of primary healthcare services; local governments are responsible for waiving administrative barriers, allocating emergency funding, and integrating returnees into existing health systems; and non-governmental organizations can provide mobile clinics, offer trauma‑informed mental health support, and conduct community outreach to identify and assist the most vulnerable. These recommendations are framed to support both the government and the returnee population while avoiding politicized attributions. They are based on lessons from global experience to promote neutral, rights-based, and effective humanitarian action. In this process of reverse migration, it is necessary to pay special attention to the health systems of the countries of origin. The lives, dignity, and future well-being of hundreds of thousands of individuals are at stake. There is a need for a call to action for the international community, regional actors, and all humanitarian organizations. Addressing this situation is not only an act of charity but a fundamental step towards fostering stability, upholding human dignity, and preventing a more widespread public health disaster in Afghanistan. The responsibility to heal this condition is, unequivocally, a shared one.

## Data Availability

No datasets were generated or analysed during the current study.
